# Performance of Serum-Based Non-Invasive Fibrosis Scores Compared with Liver Biopsy in Patients with Chronic Hepatitis B

**DOI:** 10.3390/medicina62040646

**Published:** 2026-03-28

**Authors:** Umut Devrim Binay, Faruk Karakeçili, Orçun Barkay, Betül Kuru

**Affiliations:** Department of Infectious Diseases and Clinical Microbiology, Faculty of Medicine, Erzincan Binali Yıldırım University, 24100 Erzincan, Türkiye

**Keywords:** chronic hepatitis B, liver fibrosis, non-invasive fibrosis scores, APRI, FIB-4, liver biopsy, histological activity

## Abstract

*Background and Objectives*: Accurate assessment of liver fibrosis is essential for treatment decisions in patients with chronic hepatitis B (CHB). Although liver biopsy is considered the reference standard, its invasive nature limits routine use. Serum-based non-invasive fibrosis scores have been proposed as alternatives; however, their diagnostic performance in CHB remains variable. This study aimed to compare multiple serum-based non-invasive fibrosis scores with liver biopsy findings and to evaluate their association with histological activity. *Materials and Methods*: This retrospective cross-sectional study included 219 adult patients with CHB who underwent liver biopsy with simultaneous laboratory evaluation. Patients with viral co-infections (HIV, HCV, or HDV), metabolic syndrome, diabetes mellitus, hepatic steatosis, or incomplete data were excluded. Non-invasive fibrosis scores—including APRI, FIB-4, AST/ALT ratio (AAR), age–platelet index (API), GGT-to-platelet ratio (GPR), Lok index, modified Forns index, Albumin–Bilirubin (ALBI) score, and red cell distribution width (RDW)-based indices—were calculated using routine laboratory parameters. Histopathological fibrosis staging served as the reference standard. Diagnostic performance was evaluated using receiver operating characteristic (ROC) curve analysis, and areas under the curve (AUC) were compared using the DeLong test. Associations with histological activity index (HAI) were assessed using Spearman correlation. *Results*: For the prediction of significant fibrosis (≥F2), FIB-4 demonstrated the highest AUC, followed by ALBI and APRI. For advanced fibrosis (≥F3), FIB-4 again showed the highest AUC, followed by APRI and GPR. For significant fibrosis (≥F2), DeLong analysis revealed no statistically significant differences between FIB-4 and the other serum-based scores (*p* > 0.05). APRI (*r* = 0.556, *p* < 0.001) and FIB-4 (*r* = 0.463, *p* < 0.001) showed the strongest correlations with HAI. In ROC analysis for moderate-to-severe histological activity (HAI ≥ 4), APRI demonstrated the highest diagnostic accuracy (AUC = 0.677). *Conclusions*: Serum-based non-invasive fibrosis scores demonstrate comparable but overall modest diagnostic performance for biopsy-confirmed fibrosis in patients with chronic hepatitis B. Indices such as FIB-4 and APRI demonstrated relatively better discrimination and may be considered as screening or rule-out tools in selected clinical contexts. APRI and FIB-4 also show associations with histological activity; however, their clinical application should be interpreted with caution, given their moderate discriminatory capacity.

## 1. Introduction

Chronic hepatitis B (CHB) remains a major cause of liver-related morbidity and mortality worldwide. Progressive liver fibrosis and cirrhosis substantially increase the risk of hepatocellular carcinoma and liver-related complications, making accurate fibrosis assessment a cornerstone of clinical management [1,2].

Liver biopsy has long been considered the reference standard for evaluating liver fibrosis and necroinflammatory activity [2,3]. However, its invasive nature, risk of complications, sampling variability, and limited patient acceptance restrict routine use, particularly for repeated assessments. Consequently, there has been increasing interest in non-invasive methods that could reduce the need for biopsy [2,4,5,6].

Serum-based non-invasive fibrosis scores are attractive due to their accessibility, low cost, and reproducibility [5,6,7]. Numerous studies and meta-analyses have evaluated the diagnostic performance of commonly used indices such as APRI, FIB-4, and GPR in chronic hepatitis B, generally demonstrating modest to acceptable discriminatory ability for significant and advanced fibrosis [8,9,10,11,12,13]. Previous systematic reviews and meta-analyses evaluating non-invasive fibrosis markers in chronic hepatitis B have reported that simple serum-based indices such as APRI and FIB-4 provide only modest diagnostic performance overall [13,14,15]. More recently, an individual-patient-data meta-analysis by Johannessen et al. confirmed the overall moderate accuracy of widely used serum-based fibrosis markers across diverse CHB populations [13], while the updated WHO evidence synthesis further supported the use of non-invasive tests primarily within structured diagnostic algorithms rather than as standalone definitive tools [16].

More recent studies have continued to compare biopsy with non-invasive fibrosis markers and to explore novel or composite scoring systems [17,18,19,20]. While these investigations reinforce the clinical relevance of non-invasive approaches, most report comparable performance across indices, without clear superiority of a single marker.

Nevertheless, direct head-to-head evaluations incorporating both fibrosis staging and histological inflammatory activity within the same well-characterized biopsy-referenced cohort remain relatively limited. In addition, the potential modifying effect of necroinflammatory activity on fibrosis score performance has not been comprehensively examined.

Therefore, this study aimed to perform a comprehensive comparison of multiple serum-based non-invasive fibrosis scores against biopsy-confirmed fibrosis in patients with CHB and to determine their association with histopathological findings, including both fibrosis stage and histological activity index (HAI). By simultaneously evaluating fibrosis and inflammatory activity within a homogeneous CHB cohort, we sought to provide clinically relevant data that may assist in selecting the most informative non-invasive indices for routine practice.

## 2. Materials and Methods

### 2.1. Study Design and Patient Population

This retrospective cross-sectional study was conducted in a cohort of patients undergoing follow-up for chronic hepatitis B at a tertiary care center. Patients with hepatitis B surface antigen (HBsAg) positivity for at least six months who underwent liver biopsy between 1 January 2013 and 15 October 2024 were retrospectively identified from our hospital’s medical records. The study population consisted of approximately 1500 patients with CHB under follow-up, from whom 219 adult patients who underwent liver biopsy during the study period were included.

### 2.2. Inclusion and Exclusion Criteria

Patients aged ≥18 years with a diagnosis of CHB who underwent liver biopsy and had simultaneous laboratory evaluation were included. Exclusion criteria were viral co-infection (HIV, HCV, or HDV), metabolic syndrome, diabetes mellitus, hepatic steatosis, patients receiving antiviral treatment at the time of liver biopsy, and incomplete clinical or laboratory data.

### 2.3. Data Collection

Demographic data, laboratory parameters, and histopathological findings were retrieved retrospectively from electronic medical records. Laboratory variables included hemoglobin, red blood cell count, red cell distribution width-coefficient of variation (RDW-CV), red cell distribution width–standard deviation (RDW-SD), platelet count, mean platelet volume, aspartate aminotransferase (AST), alanine aminotransferase (ALT), gamma-glutamyl transpeptidase (GGT), albumin, total bilirubin, alkaline phosphatase, international normalized ratio (INR), age, and sex.

### 2.4. Liver Biopsy and Histopathology

Liver biopsy was performed under ultrasound guidance by experienced interventional radiologists using a 16-gauge needle. Biopsy specimens were fixed in 10% neutral buffered formalin, embedded in paraffin, and subsequently sectioned for histological evaluation. Routine staining with hematoxylin and eosin was performed, along with additional histochemical staining using Masson’s trichrome and reticulin stains to assess fibrosis architecture. The median biopsy specimen length was 15 mm (interquartile range [IQR]: 12–23 mm), and the median number of portal tracts was 8 (IQR: 6–13.5). These parameters fall within the ranges generally considered acceptable for histological assessment of liver fibrosis. All biopsy samples were independently evaluated by two experienced liver pathologists who were blinded to the clinical and laboratory data. Final histopathological assessment was reached by consensus.

Necroinflammatory activity and fibrosis stage were assessed using the Ishak histological scoring system. According to the Ishak fibrosis staging system, fibrosis severity was classified as follows: F0, no fibrosis; F1, fibrous expansion of some portal areas with or without short fibrous septa; F2, fibrous expansion of most portal areas with or without short fibrous septa; F3, fibrous expansion of most portal areas with occasional portal-to-portal bridging; F4, fibrous expansion of portal areas with marked bridging, including portal-to-central bridging; F5, marked bridging with occasional nodule formation (incomplete cirrhosis); and F6, definite or probable cirrhosis [21,22].

### 2.5. Non-Invasive Fibrosis Scores

Non-invasive scores were calculated using laboratory values obtained at the time of biopsy. Upper limits of normal were defined as AST 35 U/L and GGT 38 U/L. The formulas used for AST–Platelet Ratio Index (APRI), Fibrosis-4 Index (FIB-4), AST/ALT Ratio (AAR), Age–Platelet Index (API), GGT-to-Platelet Ratio (GPR), Lok index, modified Forns index, Albumin–Bilirubin (ALBI) score, and RDW-based indices are detailed below [23,24,25,26]:

APRI: [AST (IU/L)/AST (ULN)/PLT (10^9^/L)] × 100

FIB-4: [Age (years) × AST (IU/L)]/[PLT (10^9^/L) × √ALT (IU/L)]

AAR: AST (IU/L)/ALT(IU/L)

API: The API score was calculated using a point-based system according to patient age and platelet count. Age and platelet count were categorized into predefined ranges, and the total score was obtained by summing the corresponding points.

GPR: GGT (IU/L)/[GGT (IU/L) ULN) × PLT (10^9^/L)]

Lok index: [−5.56 − 0.0089 × PLT(10^9^/L) + 1.26 × AST/ALT (IU/L) + 5.27 × INR]

Modified Forns index: The modified Forns index was calculated using age, platelet count, and GGT levels. The cholesterol parameter included in the original Forns index was omitted due to unavailable data, and the modified version was used accordingly.

ALBI: [log bilirubin (μmol/L) × 0.66) + Albumin (g/L) × −0.085

RDW-CV/Platelet ratio = RDW-CV (%)/Platelet count (10^9^/L)

RDW-SD/Platelet ratio = RDW-SD (fL)/Platelet count (10^9^/L)

#### Outcomes

Fibrosis outcomes were defined as mild fibrosis (F0–F1); significant fibrosis (≥F2); advanced fibrosis (≥F3). Histological activity outcome was defined as moderate-to-severe activity: HAI ≥ 4.

### 2.6. Statistical Analysis

Continuous variables were expressed as mean ± standard deviation or median (minimum–maximum), as appropriate, and categorical variables as counts and percentages. Diagnostic performance of non-invasive scores for significant (≥F2) and advanced fibrosis (≥F3) was assessed using ROC curve analysis, with AUCs compared using the DeLong test. Associations with HAI were evaluated using Spearman correlation. Statistical significance was defined as *p* < 0.05. Analyses were performed using Statistical Package for the Social Sciences (SPSS 25, IBM Corp., Armonk, NY, USA) package program.

Sample size considerations: This was a retrospective study; therefore, the sample size was determined by the number of eligible patients with paired liver biopsy and concurrent laboratory data during the study period. Sample adequacy was evaluated based on the precision of ROC AUC estimates (95% confidence intervals) for the main fibrosis endpoints. The analysis included 113 patients with significant fibrosis (≥F2) and 106 with mild fibrosis (F0–F1), and 51 patients with advanced fibrosis (≥F3) and 168 with <F3, providing AUC estimates with clinically interpretable confidence interval widths for the primary comparisons.

### 2.7. Ethics Approval

The study was conducted in accordance with the Declaration of Helsinki and approved by the Erzincan Binali Yildirim University Clinical Research Ethics Committee (Decision no: 2024-14/09, Date: 24 October 2024).

## 3. Results

### 3.1. Patient Characteristics

Overall, the median age was 46.0 years (IQR 34.0–59.0) and 136 (62.1%) patients were male. Median AST, ALT, and GGT values were 29.0, 36.0, and 23.9 IU/L, respectively, while median platelet count was 217 × 10^9^/L (Table 1). Laboratory parameters showed trends across fibrosis groups, with higher aminotransferase levels and lower platelet counts in patients with advanced fibrosis.

A total of 219 patients with available fibrosis staging were included in the final analysis. Baseline demographic and laboratory characteristics of the study population are summarized in Table 1. Fibrosis staging revealed mild fibrosis (F0–F1) in 106 patients (48.4%), significant fibrosis (≥F2) in 113 patients (51.6%), and advanced fibrosis (≥F3) in 51 patients (23.3%).

### 3.2. Distribution of Non-Invasive Fibrosis Scores

The distributions of serum-based non-invasive fibrosis scores across histopathological fibrosis stages are summarized in Table 2. Median values of APRI, FIB-4, GPR, and Lok index increased progressively with advancing fibrosis stage. Patients with advanced fibrosis (≥F3) demonstrated markedly higher median GPR and Lok index values compared with those with mild fibrosis (F0–F1). In contrast, AAR and API displayed limited separation between fibrosis stages. RDW-based indices manifested increasing trends with fibrosis severity but showed wider interquartile ranges.

### 3.3. Diagnostic Performance for Fibrosis

**Figure 1:**
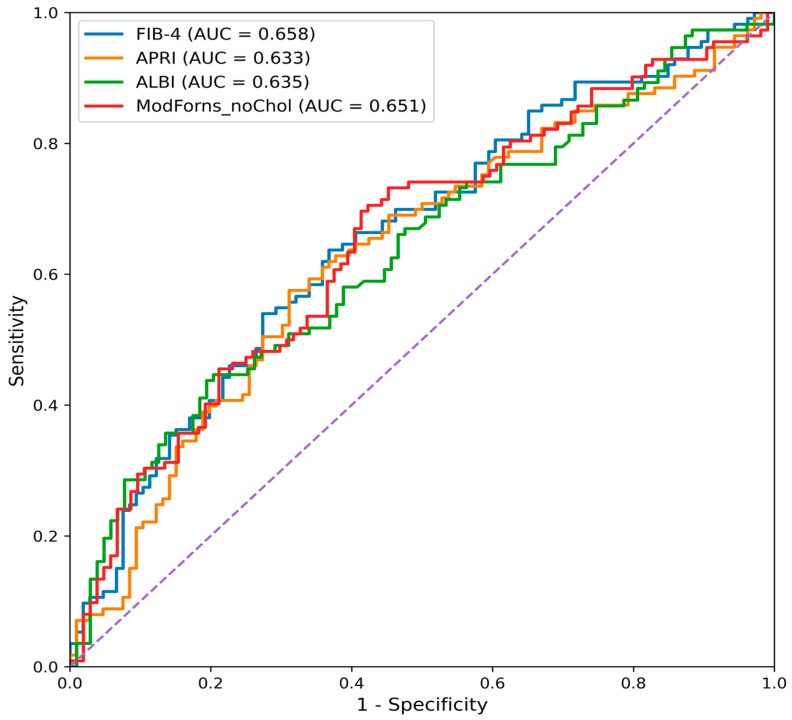
ROC curves for prediction of significant fibrosis (≥F2).

**Figure 2:**
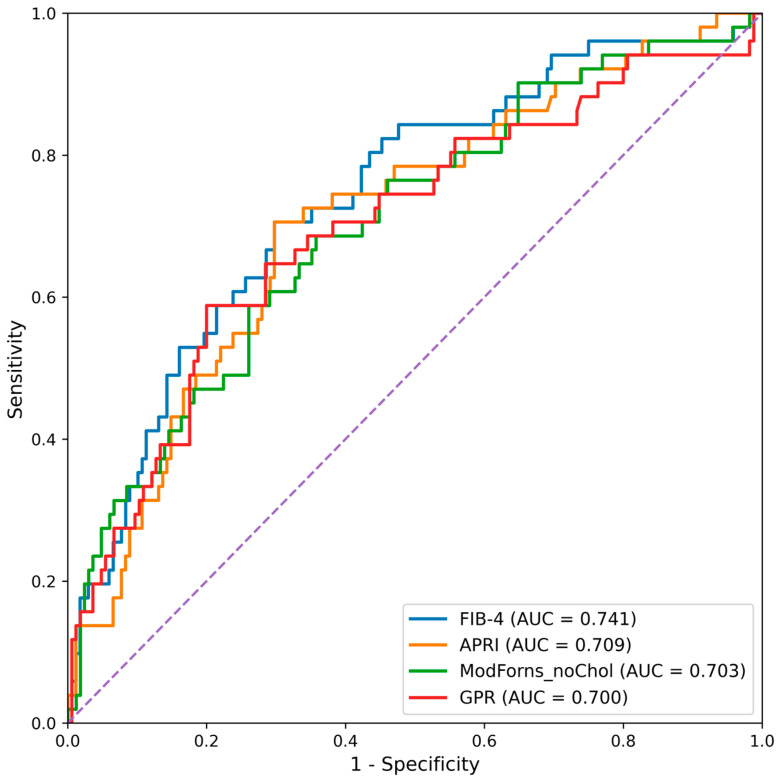
ROC curves for prediction of advanced fibrosis (≥F3).

Receiver operating characteristic (ROC) curve analysis revealed that several serum-based non-invasive fibrosis scores provided acceptable discrimination for biopsy-confirmed fibrosis. For the prediction of significant fibrosis (≥F2), FIB-4 attained the highest AUC, followed by ALBI and APRI. For advanced fibrosis (≥F3), FIB-4 again yielded the highest AUC, followed by APRI and GPR. RDW-based indices and AAR exhibited lower discriminative ability compared with established fibrosis scores (Table 3, Figure 1 and Figure 2).

Comparative ROC analysis using the DeLong test for significant fibrosis (≥F2) revealed no statistically significant differences between FIB-4 and the other serum-based scores (Table 4, *p* > 0.05).

Optimal cut-off values and diagnostic performance metrics (sensitivity, specificity, PPV, NPV, and accuracy) for all scores are provided in Table 5 and Table 6.

### 3.4. Association Between Histological Activity Index and Non-Invasive Scores

Spearman correlation analysis indicated significant associations between HAI and several non-invasive scores. The strongest correlations were observed for APRI (*r* = 0.556, *p* < 0.001) and FIB-4 (*r* = 0.463, *p* < 0.001), followed by ALBI, GPR, and modified Forns index.

**Figure 3:**
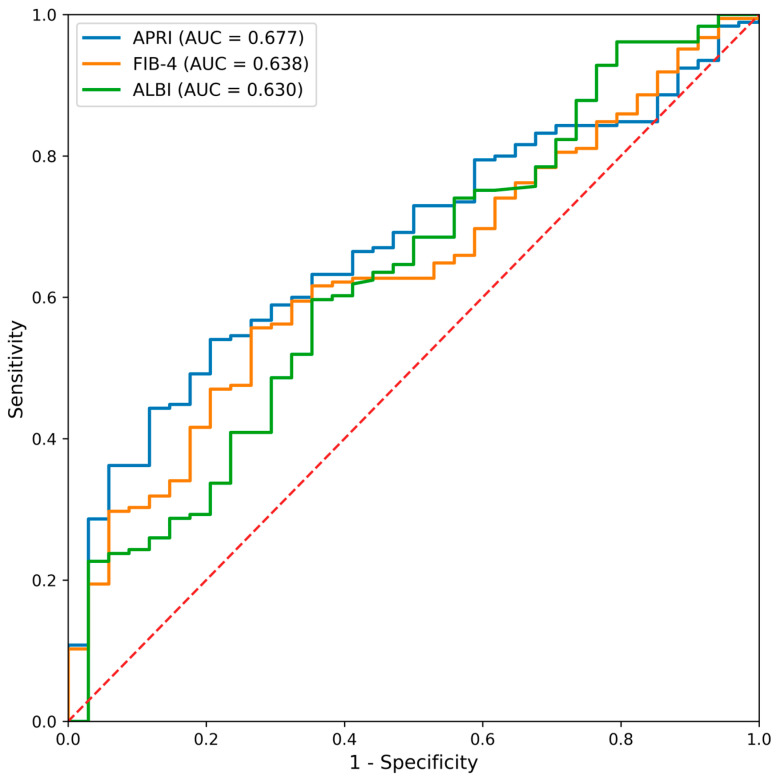
Receiver operating characteristic curves of serum-based non-invasive scores for predicting moderate-to-severe histological activity (HAI ≥ 4) in patients with chronic hepatitis B. APRI demonstrated the highest area under the curve, followed by FIB-4 and ALBI.

ROC analysis for prediction of moderate-to-severe histological activity (HAI ≥ 4) disclosed that APRI achieved the highest diagnostic accuracy (AUC = 0.677), followed by FIB-4 and ALBI (Figure 3). Detailed cut-off values and diagnostic performance metrics are presented in Table 7.

### 3.5. Influence of Histological Activity on Fibrosis Score Performance

Stratified ROC analyses according to histological activity demonstrated a marked interaction between necroinflammatory burden and fibrosis score performance. In patients with low inflammatory activity (HAI < 4), both APRI and FIB-4 showed very limited discriminatory ability for detecting significant fibrosis (≥F2), with AUC values below 0.35. For advanced fibrosis (≥F3), ROC analysis was not considered statistically reliable in this subgroup due to the extremely small number of events (n = 1). In contrast, in patients with moderate-to-severe activity (HAI ≥ 4), the diagnostic performance improved substantially, with AUC values reaching 0.695 for APRI and 0.740 for FIB-4 in the detection of advanced fibrosis (≥F3) (Table 8).

## 4. Discussion

In this retrospective cross-sectional study, we compared biopsy-confirmed fibrosis with a broad panel of serum-based non-invasive indices in adults with chronic hepatitis B. Although several comparative studies have previously assessed non-invasive fibrosis scores in chronic hepatitis B, our study adds to the existing literature by simultaneously evaluating a broad panel of established and exploratory indices against both biopsy-confirmed fibrosis and histological activity in a relatively large and homogeneous CHB cohort. The concurrent evaluation of fibrosis stage and HAI highlights the dual diagnostic potential of commonly used scores, which has been insufficiently explored in prior studies. Our main finding is that commonly used indices—including FIB-4, APRI, and GPR—demonstrated comparable diagnostic performance for identifying both significant and advanced fibrosis, with FIB-4 showing the highest area under the curve (AUC) values in our cohort [2,6,8,9].

FIB-4 showed the highest discriminative accuracy for both ≥F2 and ≥F3 fibrosis endpoints, followed by APRI and GPR, particularly for advanced fibrosis. Although numerical differences in AUC values were observed, DeLong testing did not demonstrate statistically significant differences among the evaluated scores. This finding is consistent with previous studies showing that commonly used serum-based indices provide broadly comparable discrimination in chronic hepatitis B and that no single marker consistently outperforms others across different cohorts [8,10,11,12].

Analysis of Youden-derived cut-off values revealed distinct sensitivity and specificity trade-offs across indices. Although several indices reached statistical significance, the AUC values observed in our cohort predominantly fall within a range generally considered to reflect modest discriminatory ability. From a clinical perspective, such performance limits their suitability as standalone diagnostic instruments for individual patient decision-making. However, the relatively high negative predictive values observed for APRI and FIB-4 in advanced fibrosis suggest that these scores may be more appropriately used as rule-out tools, helping to identify patients at low probability of advanced disease. Their role may therefore be best positioned within stepwise diagnostic algorithms or in resource-limited settings where access to elastography or biopsy is restricted [2,6,27]. It should also be acknowledged that liver stiffness assessment using elastography-based techniques has emerged as a major non-invasive modality for fibrosis evaluation in both chronic viral hepatitis and metabolic-associated fatty liver disease. Liver stiffness measurement has been shown to provide higher diagnostic accuracy than serum-based indices for staging significant and advanced fibrosis, as demonstrated in prior comparative analyses [28]. Accordingly, elastography is increasingly integrated into contemporary diagnostic algorithms. In this context, serum-based scores may be best viewed as complementary tools rather than replacements for imaging-based assessment.

In contrast, the age–platelet index (API) and Lok index demonstrated relatively higher specificity but lower sensitivity, especially for advanced fibrosis. This pattern suggests that these indices may be more suitable for confirming, rather than screening for, advanced disease. The limited performance of the Lok index in our cohort may be partly explained by the low proportion of decompensated patients and the relative preservation of INR values in early and intermediate stages of chronic hepatitis B [6,12,27,29].

The modified Forns index demonstrated stable AUC values for both significant and advanced fibrosis despite the exclusion of the cholesterol parameter. The combination of age, platelet count, and GGT included in this index, together with its significant correlation with histological activity, suggests that it may reflect a biological profile in which fibrosis and necroinflammation are concurrently captured [6,12,27,29].

Another finding of this study that deserves discussion is the significant association between non-invasive fibrosis scores and histological activity. APRI and FIB-4 demonstrated moderate-to-strong correlations with the histological activity index (HAI) and showed the highest diagnostic accuracy for predicting moderate-to-severe necroinflammatory activity (HAI ≥ 4). These findings suggest that commonly used aminotransferase- and platelet-based indices may reflect ongoing necroinflammatory processes in addition to structural fibrosis, likely due to their incorporation of transaminase levels that are influenced by hepatic inflammation [8,9,29]. However, the observed correlation between APRI/FIB-4 and histological activity (HAI) must be interpreted with caution. Because AST and ALT are direct biochemical markers of necroinflammation and are mathematically embedded within the APRI and FIB-4 formulas, an association with HAI is, at least in part, structurally expected. This represents a form of mathematical coupling rather than an entirely independent predictive relationship. To further explore this issue, we performed stratified analyses according to HAI categories. Notably, the discriminatory performance of aminotransferase-based indices for fibrosis detection was substantially reduced in patients with low inflammatory activity, whereas it improved in those with higher HAI scores. These findings support the interpretation that part of the apparent diagnostic performance of APRI and FIB-4 may be driven by concurrent inflammatory activity rather than fibrosis severity alone.

ALBI and GPR also demonstrated meaningful associations with both fibrosis stage and histological activity. Given that these scores incorporate parameters related to hepatic synthetic function, cholestasis, and portal hypertension, their association with histological activity is biologically plausible and has been reported in recent chronic hepatitis B cohorts [6,9,10]. It should be noted that the optimal cut-off values identified for GPR in our cohort (0.002 for ≥F2 and 0.004 for ≥F3) were numerically very low. Although these thresholds were statistically derived using the Youden index, such small absolute values may limit clinical interpretability and could be sensitive to minor laboratory variations or rounding effects. Therefore, while GPR demonstrated moderate discriminatory performance comparable to other indices, its practical applicability in routine clinical decision-making may be constrained by the instability of these thresholds. Further validation in independent cohorts using standardized measurement protocols would be necessary before recommending GPR as a standalone tool. In contrast, the AST/ALT ratio showed limited diagnostic utility for both fibrosis staging and histological activity, consistent with prior reports indicating that this ratio lacks sufficient discriminatory capacity when used as a standalone marker [9,10].

RDW-based indices were included as exploratory markers. Although statistically significant associations with fibrosis and histological activity were observed, their overall discriminative performance was inferior to that of established serum-based scores. This finding suggests that RDW-based indices are unlikely to serve as independent clinical decision tools but may have potential value as adjunctive components in future composite or hybrid scoring systems rather than as standalone diagnostic markers [30,31].

Our findings are broadly consistent with prior studies and meta-analyses demonstrating moderate diagnostic accuracy of serum-based fibrosis indices in chronic hepatitis B [8,9,10,11,12,13,14,15,16,17,18,19,20,32]. This pattern is consistent with earlier meta-analytic evidence indicating that APRI and FIB-4 offer limited-to-moderate discrimination for HBV-related fibrosis, while more complex biomarker panels and elastography-based methods may perform better in certain diagnostic contexts [13,14,15]. The individual-patient-data meta-analysis by Johannessen et al. reported AUC values within a similar range for APRI and FIB-4, reinforcing the observation that these indices provide clinically useful but imperfect discrimination [13]. In addition, the recent WHO evidence synthesis by Liguori et al. supports the view that non-invasive fibrosis scores are most appropriately interpreted within structured diagnostic pathways rather than as standalone tools [16]. Recent cohort studies and comparative analyses have likewise concluded that serum-based scores perform comparably to one another and are most appropriately used within structured diagnostic algorithms rather than as standalone tools [17,18,19,20]. Our results align with this evidence base. The incremental contribution of the present study lies in the comprehensive head-to-head comparison of a broad panel of established and exploratory indices within a homogeneous biopsy-referenced CHB cohort, and in the demonstration that necroinflammatory activity substantially modifies the apparent diagnostic performance of aminotransferase-based scores. This latter finding may help refine clinical interpretation of APRI and FIB-4 in patients with differing inflammatory states.

The relatively modest sensitivity, specificity, and predictive values observed in this study likely reflect the use of liver biopsy as the reference standard and the inclusion of intermediate fibrosis stages, which constitute a diagnostic gray zone in chronic hepatitis B. Unlike studies comparing extreme fibrosis stages or relying primarily on elastography-based reference standards, our cohort reflects real-world clinical practice and may therefore provide a more realistic estimate of the performance of serum-based non-invasive scores in routine care [2,11].

Several limitations should be acknowledged. First and foremost, the retrospective design represents a major limitation of this study. Retrospective analyses are inherently subject to selection bias, as only patients who underwent liver biopsy were included, and unmeasured confounding factors may have influenced the observed associations. Although the overall cohort size provided reasonably precise AUC estimates for the primary endpoints, the study may have been underpowered to detect small differences between AUCs in DeLong comparisons, particularly for advanced fibrosis, where the number of ≥F3 cases was limited. Therefore, non-significant DeLong test results should not be interpreted as evidence of equivalence between scores, but rather as an inability to demonstrate statistically detectable differences within the available sample. In addition, elastography-based assessments were not analyzed because these modalities were not routinely available at our institution during the study period, preventing standardized retrospective evaluation. Furthermore, patients with metabolic syndrome, diabetes mellitus, and hepatic steatosis were excluded to minimize potential confounding effects on transaminase levels and fibrosis score calculations. While this approach allowed a more focused evaluation of score performance in CHB-related liver injury, it may reduce the applicability of our results to real-world CHB populations, in which metabolic comorbidities are common. The diagnostic performance of indices such as APRI and FIB-4 may differ in patients with concomitant metabolic dysfunction. Another important limitation is the lack of long-term clinical follow-up data. Because this study was designed as a retrospective cross-sectional analysis, we were unable to assess the prognostic value of the evaluated indices in relation to clinical outcomes such as fibrosis progression, hepatic decompensation, hepatocellular carcinoma development, or liver-related mortality. Consequently, our findings pertain primarily to diagnostic performance rather than longitudinal risk stratification. Future prospective studies incorporating outcome-based endpoints are needed to clarify the prognostic implications of these scores in chronic hepatitis B.

## 5. Conclusions

Serum-based non-invasive fibrosis scores demonstrate comparable but overall modest diagnostic accuracy for biopsy-confirmed fibrosis in patients with chronic hepatitis B. While no single score clearly outperformed others, indices such as FIB-4 and APRI showed relatively better discrimination and higher negative predictive values for advanced fibrosis. These findings suggest that such scores may be most appropriately applied as screening or rule-out tools, rather than as definitive diagnostic instruments. Our findings additionally suggest that the diagnostic performance of aminotransferase-based indices may vary according to necroinflammatory activity, underscoring the importance of interpreting these scores within the broader clinical and biochemical context. Their clinical utility may be particularly relevant in resource-limited settings or as part of stepwise diagnostic strategies, whereas confirmatory evaluation with elastography or biopsy remains necessary when precise staging is required.

## Figures and Tables

**Table 1:** Baseline demographic and laboratory characteristics of the study population.

	Overall	F0–F1	≥F2	≥F3
**N**	219	106	113	51
**Male, n (%)**	136 (62.1%)	63 (59.4%)	40 (35.4%)	33 (64.7%)
**Age (years)**	46.00 (34.00–59.00)	42.00 (31.25–53.50)	48.50 (35.00–60.75)	51.00 (41.00–64.50)
**AST (IU/L)**	29.00 (22.00–49.50)	26.00 (21.00–38.50)	30.00 (21.00–51.25)	42.00 (27.50–73.50)
**ALT (IU/L)**	36.00 (21.00–77.50)	30.00 (20.00–51.00)	37.50 (19.25–76.00)	56.00 (28.50–104.00)
**GGT (IU/L)**	23.90 (16.00–36.00)	21.50 (15.17–29.45)	23.80 (18.00–31.00)	32.00 (22.00–52.40)
**Platelets (10^9^/L)**	217.00 (186.50–259.00)	222.00 (193.00–275.00)	227.50 (197.00–262.00)	193.00 (171.50–230.50)
**Albumin (g/L)**	42.00 (40.00–44.62)	43.20 (41.20–45.05)	42.00 (40.28–44.38)	40.10 (37.55–43.00)
**Total bilirubin (mg/dL)**	0.68 (0.48–0.90)	0.69 (0.50–0.91)	0.61 (0.46–0.83)	0.77 (0.47–1.00)
**ALP (IU/L)**	80.00 (65.00–96.50)	76.00 (65.00–93.00)	82.00 (64.75–97.75)	85.00 (66.50–109.50)
**INR**	1.07 (1.00–1.17)	1.06 (0.98–1.13)	1.06 (1.00–1.17)	1.13 (1.04–1.23)
**Hemoglobin (g/dL)**	15.20 (13.60–16.00)	15.20 (13.80–16.10)	15.05 (13.40–15.97)	15.20 (14.00–16.00)
**RDW-CV (%)**	13.00 (12.40–13.80)	12.85 (12.30–13.67)	13.00 (12.50–13.60)	13.10 (12.50–14.20)
**RDW-SD (fL)**	40.10 (38.00–42.70)	39.65 (37.90–41.88)	40.95 (38.32–43.55)	40.80 (37.45–44.60)

Data are presented as median (interquartile range) for continuous variables and number (percentage) for categorical variables. ALT: Alanine Aminotransferase, AST: Aspartate Aminotransferase, GGT: Gamma-Glutamyl Transferase, ALP: Alkaline Phosphatase, RDW-CV: Red Cell Distribution Width–Coefficient of Variation, RDW-SD: Red Cell Distribution Width–Standard Deviation, F: Fibrosis.

**Table 2:** Distribution of serum-based non-invasive fibrosis scores according to histopathological fibrosis stage.

Score	Fibrosis Group	Median	IQR	N
**APRI**	F0–F1	0.333	0.24–0.537	106
**APRI**	≥F2	0.409	0.262–0.656	113
**APRI**	≥F3	0.651	0.406–1.244	51
**FIB-4**	F0–F1	0.937	0.625–1.354	106
**FIB-4**	≥F2	1.078	0.751–1.427	113
**FIB-4**	≥F3	1.646	1.056–2.432	51
**AAR**	F0–F1	0.866	0.654–1.123	106
**AAR**	≥F2	0.787	0.671–1.046	113
**AAR**	≥F3	0.811	0.702–1.057	51
**API**	F0–F1	3.0	2.0–4.0	106
**API**	≥F2	3.0	2.0–5.0	113
**API**	≥F3	5.0	3.0–6.5	51
**GPR**	F0–F1	0.002	0.002–0.004	104
**GPR**	≥F2	0.003	0.002–0.004	112
**GPR**	≥F3	0.005	0.003–0.007	51
**Lok Index**	F0–F1	0.322	0.185–0.422	106
**Lok Index**	≥F2	0.335	0.213–0.423	112
**Lok Index**	≥F3	0.416	0.245–0.595	50
**ALBI**	F0–F1	−2.967	−3.147–−2.813	103
**ALBI**	≥F2	−2.907	−3.09–−2.699	112
**ALBI**	≥F3	−2.743	−2.947–−2.417	51
**RDW-CV/Plt**	F0–F1	0.057	0.05–0.069	106
**RDW-CV/Plt**	≥F2	0.057	0.049–0.068	113
**RDW-CV/Plt**	≥F3	0.068	0.055–0.079	51
**RDW-SD/Plt**	F0–F1	0.178	0.149–0.215	106
**RDW-SD/Plt**	≥F2	0.179	0.15–0.217	113
**RDW-SD/Plt**	≥F3	0.202	0.178–0.245	51
**Modified Forns Index**	F0–F1	6.126	5.165–7.365	104
**Modified Forns Index**	≥F2	6.735	5.43–7.694	112
**Modified Forns Index**	≥F3	7.48	6.519–8.959	51

AAR: Aspartate Aminotransferase to Alanine Aminotransferase Ratio, APRI: Aspartate Aminotransferase to Platelet Ratio Index, FIB-4: Fibrosis-4 Index, GPR: Gamma-Glutamyl Transferase to Platelet Ratio, RDW-CV: Red Cell Distribution Width–Coefficient of Variation, RDW-SD: Red Cell Distribution Width–Standard Deviation, ALBI: Albumin–Bilirubin Score, IQR: Interquartile Range, PLT: Platelet.

**Table 3:** ROC curve analysis of non-invasive fibrosis scores or prediction of significant (≥F2) and advanced fibrosis (≥F3).

Score	Fibrosis Endpoint	AUC	95% CI	N
**APRI**	≥F2	0.633	0.56–0.706	113
**APRI**	≥F3	0.709	0.622–0.796	51
**FIB-4**	≥F2	0.658	0.586–0.729	113
**FIB-4**	≥F3	0.741	0.656–0.825	51
**AAR**	≥F2	0.481	0.404–0.557	113
**AAR**	≥F3	0.507	0.416–0.598	51
**API**	≥F2	0.621	0.548–0.695	113
**API**	≥F3	0.687	0.599–0.776	51
**GPR**	≥F2	0.619	0.544–0.693	112
**GPR**	≥F3	0.7	0.612–0.787	51
**Lok Index**	≥F2	0.565	0.489–0.641	112
**Lok Index**	≥F3	0.635	0.543–0.726	50
**ALBI**	≥F2	0.635	0.561–0.709	112
**ALBI**	≥F3	0.684	0.595–0.773	51
**RDW-CV/Plt**	≥F2	0.567	0.491–0.642	113
**RDW-CV/Plt**	≥F3	0.663	0.574–0.753	51
**RDW-SD/Plt**	≥F2	0.566	0.491–0.642	113
**RDW-SD/Plt**	≥F3	0.647	0.557–0.737	51
**Modified Forns Index**	≥F2	0.651	0.578–0.723	112
**Modified Forns Index**	≥F3	0.703	0.616–0.79	51

AAR: Aspartate Aminotransferase to Alanine Aminotransferase Ratio, APRI: Aspartate Aminotransferase to Platelet Ratio Index, FIB-4: Fibrosis-4 Index, GPR: Gamma-Glutamyl Transferase to Platelet Ratio, RDW-CV: Red Cell Distribution Width–Coefficient of Variation, RDW-SD: Red Cell Distribution Width–Standard Deviation, ALBI: Albumin–Bilirubin Score, IQR: Interquartile Range, PLT: Platelet.

**Table 4:** DeLong test comparisons of serum-based non-invasive fibrosis scores for the prediction of significant fibrosis (Ishak ≥ F2).

Score	N	AUC (FIB-4, ≥F2)	AUC (Comparator, ≥F2)	AUC Diff (Ref-Score)	*p* Value
**AAR**	219	0.658	0.481	0.177	0.08
**RDW-SD/Plt**	219	0.658	0.566	0.092	0.16
**RDW-CV/Plt**	219	0.658	0.567	0.091	0.162
**Lok Index**	218	0.656	0.565	0.091	0.232
**API**	219	0.658	0.621	0.036	0.429
**GPR**	216	0.659	0.619	0.04	0.603
**APRI**	219	0.658	0.633	0.025	0.709
**ALBI**	215	0.656	0.635	0.021	0.793

AAR: Aspartate Aminotransferase to Alanine Aminotransferase Ratio, APRI: Aspartate Aminotransferase to Platelet Ratio Index, GPR: Gamma-Glutamyl Transferase to Platelet Ratio, RDW-CV: Red Cell Distribution Width–Coefficient of Variation, RDW-SD: Red Cell Distribution Width–Standard Deviation, ALBI: Albumin–Bilirubin Score, IQR: Interquartile Range, PLT: Platelet.

**Table 5:** Optimal cut-off values of serum-based non-invasive fibrosis scores for the prediction of significant fibrosis (≥F2).

Score	N	AUC	Cut-Off (Youden)	Sensitivity (%)	Specificity (%)	PPV (%)	NPV (%)	Accuracy (%)
**APRI**	219	0.633	0.447	57.5	68.9	66.3	60.3	63
**FIB-4**	219	0.658	1.032	63.7	63.2	64.9	62	63.5
**AAR**	219	0.481	0.707	71.7	35.8	54.4	54.3	54.3
**API**	219	0.621	5	40.7	81.1	69.7	56.2	60.3
**GPR**	216	0.619	0.002	69.6	51.9	60.9	61.4	61.1
**Lok Index**	218	0.565	0.332	57.1	55.7	57.7	55.1	56.4
**ALBI**	215	0.635	−2.792	43.8	80.6	71	56.8	61.4
**RDW-CV/Plt**	219	0.567	0.059	56.6	56.6	58.2	55	56.6
**RDW-SD/Plt**	219	0.566	0.19	52.2	62.3	59.6	55	57.1

AAR: Aspartate Aminotransferase to Alanine Aminotransferase Ratio, APRI: Aspartate Aminotransferase to Platelet Ratio Index, FIB-4: Fibrosis-4 Index, GPR: Gamma-Glutamyl Transferase to Platelet Ratio, RDW-CV: Red Cell Distribution Width–Coefficient of Variation, RDW-SD: Red Cell Distribution Width–Standard Deviation, ALBI: Albumin–Bilirubin Score, IQR: Interquartile Range, PLT: Platelet.

**Table 6:** Optimal cut-off values of serum-based non-invasive fibrosis scores for the prediction of advanced fibrosis (≥F3).

Score	N	AUC	Cut-Off (Youden)	Sensitivity (%)	Specificity (%)	PPV (%)	NPV (%)	Accuracy (%)
**APRI**	219	0.709	0.498	70.6	70.2	41.9	88.7	70.3
**FIB-4**	219	0.741	1.295	70.6	70.2	41.9	88.7	70.3
**AAR**	219	0.507	0.732	72.5	39.9	26.8	82.7	47.5
**API**	219	0.687	5	56.9	78	43.9	85.6	73.1
**GPR**	216	0.7	0.004	58.8	80	47.6	86.3	75
**Lok Index**	218	0.635	0.412	52	72	35.6	83.4	67.4
**ALBI**	215	0.684	−2.786	56.9	76.2	42.6	85	71.6
**RDW-CV/Plt**	219	0.663	0.06	72.5	57.7	34.3	87.4	61.2
**RDW-SD/Plt**	219	0.647	0.187	68.6	59.5	34	86.2	61.6

AAR: Aspartate Aminotransferase to Alanine Aminotransferase Ratio, APRI: Aspartate Aminotransferase to Platelet Ratio Index, FIB-4: Fibrosis-4 Index, GPR: Gamma-Glutamyl Transferase to Platelet Ratio, RDW-CV: Red Cell Distribution Width–Coefficient of Variation, RDW-SD: Red Cell Distribution Width–Standard Deviation, ALBI: Albumin–Bilirubin Score, IQR: Interquartile Range, PLT: Platelet.

**Table 7:** Optimal cut-off values of serum-based non-invasive fibrosis scores according to HAI.

Score	N	AUC	Cut-Off (Youden)	Sensitivity (%)	Specificity (%)	PPV (%)	NPV (%)	Accuracy (%)	Spearman r	*p* Value
**APRI**	219	0.677	0.411	54.1	79.4	93.5	24.1	58	0.556	0.000
**FIB-4**	219	0.638	1.025	55.7	73.5	92	23.4	58.4	0.463	0.000
**ALBI**	215	0.63	−2.944	59.7	64.7	90	23.2	60.5	0.397	0.000
**Lok Index**	218	0.628	0.298	61.4	67.6	91.1	24.5	62.4	0.365	0.000
**RDW-CV/Plt**	219	0.621	0.052	74.1	50	89	26.2	70.3	0.361	0.000
**Modified Forns Index**	216	0.593	6.735	52.2	73.5	91.3	22.3	55.6	0.302	0.000
**RDW-SD/Plt**	219	0.591	0.19	48.6	73.5	90.9	20.8	52.5	0.289	0.000
**API**	219	0.579	5	34.1	91.2	95.5	20.3	42.9	0.241	0.0003
**GPR**	216	0.566	0.004	34.1	88.2	93.9	20	42.6	0.194	0.004
AAR	219	0.479	1.286	13	94.1	92.3	16.6	25.6	−0.175	0.0094

AAR: Aspartate Aminotransferase to Alanine Aminotransferase Ratio, APRI: Aspartate Aminotransferase to Platelet Ratio Index, FIB-4: Fibrosis-4 Index, GPR: Gamma-Glutamyl Transferase to Platelet Ratio, RDW-CV: Red Cell Distribution Width–Coefficient of Variation, RDW-SD: Red Cell Distribution Width–Standard Deviation, ALBI: Albumin–Bilirubin Score, IQR: Interquartile Range, PLT: Platelet.

**Table 8:** Stratified ROC analysis of APRI and FIB-4 according to histological activity.

HAI Group	Fibrosis Endpoint	N (Total)	N (Event)	APRI (AUC)	FIB-4 (AUC)
HAI < 4	≥F2	34	7	0.238	0.317
HAI < 4	≥F3	34	1	Not reliable *	Not reliable *
HAI ≥ 4	≥F2	185	106	0.639	0.675
HAI ≥ 4	≥F3	185	50	0.695	0.740

* ROC analysis for ≥F3 in the HAI < 4 subgroup was not considered reliable due to the extremely limited number of advanced fibrosis cases (n = 1), rendering AUC estimates statistically unstable.

## Data Availability

The data presented in this study are available on request from the corresponding author.

## Abbreviations

The following abbreviations are used in this manuscript:

AAR	Aspartate Aminotransferase to Alanine Aminotransferase Ratio
AASLD	American Association for the Study of Liver Diseases
ALBI	Albumin–Bilirubin Score
ALT	Alanine Aminotransferase
APRI	Aspartate Aminotransferase to Platelet Ratio Index
AST	Aspartate Aminotransferase
AUC	Area Under the Curve
CHB	Chronic Hepatitis B
CI	Confidence Interval
DM	Diabetes Mellitus
EASL	European Association for the Study of the Liver
FIB-4	Fibrosis-4 Index
GGT	Gamma-Glutamyl Transferase
GPR	Gamma-Glutamyl Transferase to Platelet Ratio
HAI	Histological Activity Index
HBV	Hepatitis B Virus
HCC	Hepatocellular Carcinoma
HCV	Hepatitis C Virus
HDV	Hepatitis D Virus
HIV	Human Immunodeficiency Virus
INR	International Normalized Ratio
IQR	Interquartile Range
ISHAK	Ishak Histological Scoring System
METAVIR	METAVIR Fibrosis Scoring System
MPV	Mean Platelet Volume
NPV	Negative Predictive Value
PLT	Platelet Count
PPV	Positive Predictive Value
RDW	Red Cell Distribution Width
RDW-CV	Red Cell Distribution Width–Coefficient of Variation
RDW-SD	Red Cell Distribution Width–Standard Deviation
ROC	Receiver Operating Characteristic
SPSS	Statistical Package for the Social Sciences
ULN	Upper Limit of Normal
WHO	World Health Organization

## References

[1] World Health Organization (2024). Guidelines for the Prevention, Diagnosis, Care and Treatment for People with Chronic Hepatitis B Infection.

[2] Cornberg M., Sandmann L., Jaroszewicz J., Kennedy P., Lampertico P., Lemoine M., Lens S., Testoni B., Wong G.L.-H., Russo F.P. (2025). EASL Clinical Practice Guidelines on the management of hepatitis B virus infection. J. Hepatol..

[3] Patel K., Sebastiani G. (2020). Limitations of non-invasive tests for assessment of liver fibrosis. JHEP Rep..

[4] Ghany M.G., Pan C.Q., Lok A.S., Feld J.J., Lim J.K., Wang S.H., Kim A.Y., Tang A.S., Nguyen M.H., Naggie S. (2025). AASLD/IDSA practice guideline on treatment of chronic hepatitis B. Hepatology.

[5] Lin C.-L., Kao J.-H. (2021). Novel biomarkers for the management of chronic hepatitis B virus infection. Hepatitis B Virus and Liver Disease.

[6] Bera C., Hamdan-Perez N., Patel K. (2024). Non-invasive assessment of liver fibrosis in hepatitis B patients. J. Clin. Med..

[7] Okdemir S., Cakmak E. (2022). A novel non-invasive score for the prediction of advanced fibrosis in patients with chronic hepatitis B. Ann. Hepatol..

[8] Gür-Altunay D., Yürük-Atasoy P. (2023). How successful are APRI and FIB-4 scores in predicting liver fibrosis in chronic hepatitis B patients?. Infect. Dis. Clin. Microbiol..

[9] Wang L., Li J., Yang K., Zhang H., Wang Q., Lv X., Guan S. (2020). Comparison and evaluation of non-invasive models in predicting liver inflammation and fibrosis in chronic hepatitis B virus–infected patients with high HBV DNA and normal or mildly elevated ALT levels. Medicine.

[10] Ekin N., Uçmak F., Ebik B., Tuncel E., Kaçmaz H., Arpa M., Atay A. (2022). GPR, King’s score and S-index are superior to other non-invasive fibrosis markers in predicting liver fibrosis in chronic hepatitis B patients. Acta Gastro.-Enterol. Belg..

[11] Çelik D., Tatar B., Köse Ş., Ödemiş İ. (2020). Evaluation of the diagnostic validity of noninvasive tests for predicting liver fibrosis stage in chronic hepatitis B patients. Acta Gastro.-Enterol. Belg..

[12] Addissouky T.A., Sayed I.E.T.E., Agroudy A.E.E., Wang Y. (2025). Evaluating non-invasive biomarkers and composite scores for liver fibrosis diagnosis in hepatitis B and C infections. SN Compr. Clin. Med..

[13] Johannessen A., Stockdale A.J., Henrion M.Y.R., Okeke E., Seydi M., Wandeler G., Sonderup M., Spearman C.W., Vinikoor M., Sinkala E. (2023). Systematic review and individual-patient-data meta-analysis of non-invasive fibrosis markers for chronic hepatitis B in Africa. Nat. Commun..

[14] Xu X.-Y., Kong H., Song R.-X., Zhai Y.-H., Wu X.-F., Ai W.-S., Liu H.-B. (2014). The effectiveness of noninvasive biomarkers to predict hepatitis B-related significant fibrosis and cirrhosis: A systematic review and meta-analysis of diagnostic test accuracy. PLoS ONE.

[15] Houot M., Ngo Y., Munteanu M., Marque S., Poynard T. (2016). Systematic review with meta-analysis: Direct comparisons of biomarkers for the diagnosis of fibrosis in chronic hepatitis C and B. Aliment. Pharmacol. Ther..

[16] Liguori A., Zoncapè M., Casazza G., Easterbrook P., Tsochatzis E.A. (2025). Staging liver fibrosis and cirrhosis using non-invasive tests in people with chronic hepatitis B to inform WHO 2024 guidelines: A systematic review and meta-analysis. Lancet Gastroenterol. Hepatol..

[17] Borcak D., Yesilbag Z., Ozdemir Y.E., Demir A.S., Dogdas E.S., Sezen A.I., Unlu E.C., Senoglu S., Karaosmanoglu H.K., Yasar K.K. (2025). Assessing liver fibrosis in chronic hepatitis B: Liver biopsy or non-invasive fibrosis markers?. J. Clin. Med..

[18] Tamaki N., Takaura K., Higuchi M., Yasui Y., Itakura J., Tsuchiya K., Nakanishi H., Izumi N., Kurosaki M. (2024). Enhanced liver fibrosis score for diagnosing liver fibrosis in chronic hepatitis. Diagnostics.

[19] Ozguler M., Durak S., Solmaz O.A., Eser Karlidag G., Gundag O., Kirik Y., Tanir B., Kara S.S. (2025). The use of serum scoring systems in predicting liver fibrosis caused by chronic hepatitis B: A retrospective case-control study. Medicina.

[20] Hudu S.A., Shinkafi S.H., Jimoh A.O. (2024). A critical review of diagnostic and prognostic markers of chronic hepatitis B infection. Med. Rev..

[21] Ishak K., Baptista A., Bianchi L., Callea F., De Groote J., Gudat F., Denk H., Desmet V., Korb G., MacSween R.N. (1995). Histological grading and staging of chronic hepatitis. J. Hepatol..

[22] Bedossa P., Patel K., Castera L. (2015). Histologic and noninvasive estimates of liver fibrosis. Clin. Liver Dis..

[23] Li J., Mao R., Li X., Zheng J., Qi X., Yuan Q., Zhang J., Zhang J.-M., Xia N.-S. (2018). A novel noninvasive index for the prediction of moderate to severe fibrosis in chronic hepatitis B patients. Dig. Liver Dis..

[24] Wang R.-Q., Zhang Q.-S., Zhao S.-X., Niu X.-M., Du J.-H., Du H.-J., Nan Y.-M. (2016). Gamma-glutamyl transpeptidase to platelet ratio index is a good noninvasive biomarker for predicting liver fibrosis in Chinese chronic hepatitis B patients. J. Int. Med. Res..

[25] Wu D., Rao Q., Chen W., Ji F., Xie Z., Huang K., Chen E., Zhao Y., Ouyang X., Zhang S. (2018). Development and validation of a novel score for fibrosis staging in patients with chronic hepatitis B. Liver Int..

[26] Kayadibi H., Yilmaz S., Yeniova A.O., Koseoglu H., Simsek Z. (2020). Development and evaluation of a novel noninvasive index for predicting significant fibrosis, advanced fibrosis, and cirrhosis in patients with chronic hepatitis B infection. Eur. J. Gastroenterol. Hepatol..

[27] Castera L., Rinella M.E., Tsochatzis E.A. (2025). Noninvasive assessment of liver fibrosis. N. Engl. J. Med..

[28] Facciorusso A., Del Prete V., Turco A., Buccino R.V., Nacchiero M.C., Muscatiello N. (2018). Long-term liver stiffness assessment in hepatitis C virus patients undergoing antiviral therapy: Results from a 5-year cohort study. J. Gastroenterol. Hepatol..

[29] Tsuji Y., Namisaki T., Kaji K., Takaya H., Nakanishi K., Sato S., Saikawa S., Sawada Y., Kitagawa K., Shimozato N. (2020). Comparison of serum fibrosis biomarkers for diagnosing significant liver fibrosis in patients with chronic hepatitis B. Exp. Ther. Med..

[30] Yang K., Sun B., Zhang S., Pan Y., Fang J. (2023). RDW-SD is superior to RDW-CV in reflecting liver fibrosis stage in patients with chronic hepatitis B. Infect. Drug Resist..

[31] Guller B.Y., Gulumsek E., Sumbul H.E., Avci B.S., Tas A. (2023). RDW predicts fibrosis in patients with chronic hepatitis B having persistently normal ALT levels. Ethiop. J. Health Sci..

[32] Aydın M.K. (2025). The silent face of chronic hepatitis B: Biopsy-supported fibrosis detection and the reliability of non-invasive scores (FIB-4, APRI) in inactive, gray zone, and immune-tolerant cases. Med. Sci. Monit..

